# Effects of maternal exposure to arsenic on social behavior and related gene expression in F2 male mice

**DOI:** 10.1186/s12199-021-00956-y

**Published:** 2021-03-11

**Authors:** Soe-Minn Htway, Takehiro Suzuki, Sanda Kyaw, Keiko Nohara, Tin-Tin Win-Shwe

**Affiliations:** 1 Department of Physiology, University of Medicine, Magway, Magway, Myanmar; 2 Center for Health and Environmental Risk Research, National Institute for Environmental Studies, 16-2 Onogawa, Tsukuba, 305-8506 Japan

**Keywords:** Arsenic, F2 male mice, Maternal exposure, Serotonin, Social behavior

## Abstract

**Background:**

Arsenic is a developmental neurotoxicant. It means that its neurotoxic effect could occur in offspring by maternal arsenic exposure. Our previous study showed that developmental arsenic exposure impaired social behavior and serotonergic system in C3H adult male mice. These effects might affect the next generation with no direct exposure to arsenic. This study aimed to detect the social behavior and related gene expression changes in F2 male mice born to gestationally arsenite-exposed F1 mice.

**Methods:**

Pregnant C3H/HeN mice (F0) were given free access to tap water (control mice) or tap water containing 85 ppm sodium arsenite from days 8 to 18 of gestation. Arsenite was not given to F1 or F2 mice. The F2 mice were generated by mating among control F1 males and females, and arsenite-F1 males and females at the age of 10 weeks. At 41 weeks and 74 weeks of age respectively, F2 males were used for the assessment of social behavior by a three-chamber social behavior apparatus. Histological features of the prefrontal cortex were studied by ordinary light microscope. Social behavior-related gene expressions were determined in the prefrontal cortex by real time RT-PCR method.

**Results:**

The arsenite-F2 male mice showed significantly poor sociability and social novelty preference in both 41-week-old group and 74-week-old group. There was no significant histological difference between the control mice and the arsenite-F2 mice. Regarding gene expression, serotonin receptor 5B (5-HT 5B) mRNA expression was significantly decreased (*p* < 0.05) in the arsenite-F2 male mice compared to the control F2 male mice in both groups. Brain-derived neurotrophic factor (BDNF) and dopamine receptor D1a (Drd1a) gene expressions were significantly decreased (*p* < 0.05) only in the arsenite-F2 male mice of the 74-week-old group. Heme oxygenase-1 (HO-1) gene expression was significantly increased (*p* < 0.001) in the arsenite-F2 male mice of both groups, but plasma 8-hydroxy-2′-deoxyguanosine (8-OHdG) and cyclooxygenase-2 (COX-2) gene expression were not significantly different. Interleukin-1β (IL-1β) mRNA expression was significantly increased only in 41-week-old arsenite-F2 mice.

**Conclusions:**

These findings suggest that maternal arsenic exposure affects social behavior in F2 male mice via serotonergic system in the prefrontal cortex. In this study, COX-2 were not increased although oxidative stress marker (HO-1) was increased significantly in arsnite-F2 male mice.

## Background

Arsenic is well-known to be toxic to most of the tissues in the body after direct exposure. Direct exposure may be through inhalation, ingestion, or direct skin contact with arsenic-contaminated air, food and drinks, or products [1]. Commonly, the exposure is by drinking arsenic-contaminated water. Permissible level for arsenic in drinking water is 10 ppb for most of the countries over the world. There are many evidences of health hazards due to drinking arsenic-contaminated water above this level for a certain period. Severity of health hazards varied with the level of contamination as well as duration of exposure. Types of health hazards also varied from skin lesions to cancerous lesions. Neurological lesions include peripheral neuropathy, memory loss, and behavior changes [2]. These changes are cumulatively known as neurotoxic effects of arsenic, and its effect is more distinct in the developing brain. From the evidence of animal studies, developmental arsenic exposure leads to definite health hazards in the offspring because arsenic can pass through the placenta [3]. In experimental design, developmental exposure is also known as maternal exposure because arsenic-contaminated water is only given to pregnant mother and not given directly to the offspring. Exposure occurs from mother to offspring through placenta during gestational period and so it is also called gestational exposure. In some study design, maternal exposure is given during gestation, after delivery, and during lactational period. Arsenic is never given directly to offspring, but it might reach to offspring through placenta circulation and lactation [4].

Arsenic can pass through not only the placenta barrier but also the blood-brain barrier [5]. Therefore, toxic effect is possible to adult brain, and it might be more intense for a developing brain. Being a developmental neurotoxicant, neurotoxic effects of arsenic can be seen by maternal arsenic exposure during gestational period [6, 7]. Our previous study proved that maternal arsenic exposure from gestational days 8–18 impaired the social behavior and related gene expressions in F1 mice. In that study, we concluded that the behavioral changes might be due to impaired serotonergic system [8]. Effects of maternal arsenic exposure to F1 mice were studied not only in the nervous system but also in the other systems. Nohara et al. studied the tumor-augmenting effect of arsenic on liver cells in a similar study design. They showed that tumor-augmenting effect occurred in both F1 and F2 mice by maternal arsenic exposure [9].

Although arsenic was not given to the F1 mice, they might get arsenic exposure indirectly from the mother during developing period. For the F2 mice, they were generated by mating among F1 males and females, and there was no direct as well as indirect exposure to arsenic. Etiology of cancer is related to genetics, and so the tumor-augmenting effect in F2 mice might be due to genetics in origin. In the neurotoxic study, it was uncertain to see the similar effects in the next generation. This study aimed to detect the neurotoxic effects in F2 male mice born to gestationally arsenite-exposed F1 mice. In the present study, social behavior, histological feature of the prefrontal cortex, and social behavior-related gene expressions were studied in the 41-week-old and 74-week-old groups of C3H male mice.

## Methods

### Animals

**Fig. 1:**
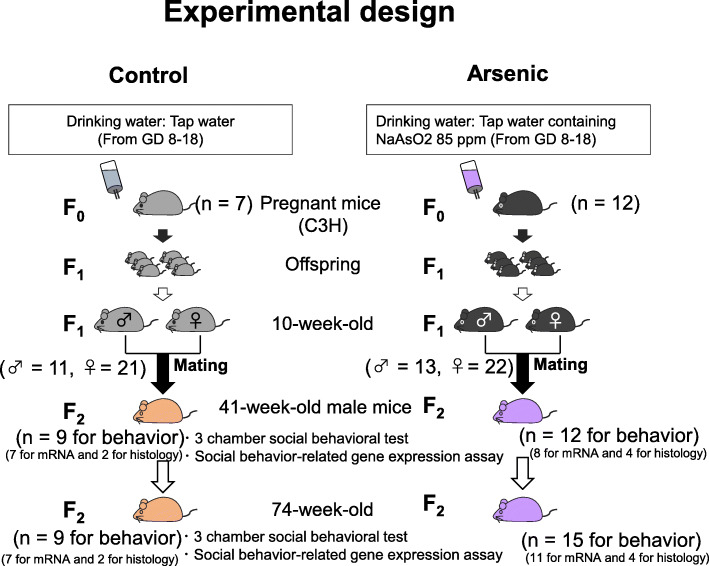
Experimental design. Pregnant C3H/HeN mice (F0) (*n* = 19) were used in this study. The arsenite mice (F0; *n* = 12) were exposed to sodium arsenite (NaAsO_2_, 85 ppm (85 mg/L) in the drinking water) from gestational days (GD) 8 to 18. The control mice (F0; *n* = 7) were given (arsenic free) tap water. The F1 male and female pups were weaned at postnatal day 21. At the age of 10 weeks, a mating was done among the control F1 males and females (1:2) to generate the control F2 mice. Similarly, to generate the arsenite-F2 mice, the arsenite-F1 males and females were mated (1:2). The F2 male pups (*n* = 18 for the control group: *n* = 27 for arsenite group) were weaned at postnatal day 21. At the age of 41 weeks, the control male mice (*n* = 9) and arsenite male mice (*n* = 12) were used for social behavior test and molecular analyses. At the age of 74 weeks, the control male mice (*n* = 9) and arsenite male mice (*n* = 15) were used for social behavior test and molecular analyses

Pregnant C3H/HeN mice (F0) (*n* = 19) were purchased from CLEA (Tokyo, Japan) and kept at 24±1 °C and 12 h light/dark cycle with free access to standard diet (CA-1; CLEA Japan). The arsenite mice (F0; *n* = 12) were exposed to sodium arsenite (NaAsO_2_, 85 ppm (85 mg/L) in the drinking water) from gestational days (GD) 8 to 18 as described previously [8, 10]. The control mice (F0; *n* = 7) were given (arsenic free) tap water. F1 offspring were 3–9 per dam in the control group and 4–8 per dam in the arsenic group. The F1 male and female pups were weaned at postnatal day 21. At the age of 10 weeks, a mating was done among the control F1 males and females (1:2) to generate the control F2 mice. Similarly, to generate the arsenite-F2 mice, the arsenite-F1 males and females mated (1:2). The F2 male pups (*n* = 18 for the control group: *n* = 27 for arsenite group) were weaned at postnatal day 21. At the age of 41 weeks, the control male mice (*n* = 9) and arsenite male mice (*n* = 12) were used for social behavior test and molecular analyses. At the age of 74 weeks, the control male mice (*n* = 9) and arsenite male mice (*n* = 15) were used for social behavior test and molecular analyses. The number of animals used is expressed in Fig. 1. The experimental protocols were approved by the Ethics Committee of the Animal Care and Experimentation Council of the National Institute for Environmental Studies (NIES), Japan (ethics approval code number: AE-19-26).

### Social behavior

Social behavior tasks were examined at the age of 41 weeks for one group and at the age of 74 weeks for the other group in F2 male mice, using a three-chambered social behavior apparatus (Muromachi Kikai Co. Ltd., Tokyo, Japan) and the ANY-maze software (Stoelting Co., Wood Dale, IL, USA). Three-chambered Plexiglas box (100 cm × 100 cm × 35 cm) purchased from Muromachi Kikai was used as described previously [11]. The box was divided into three equal chambers by transparent partition with a square-shaped hole (10 cm × 10 cm) at the base, allowing free access to all chambers. Social behavior test consists of three phases including a habituation phase for 5 min, a sociability phase for 10 min and a social novelty preference for 10 min. First of all, a subject mouse was placed in the middle chamber and allowed 5 min for habituation while an empty wired cup (15 cm in diameter and 30-cm height) was placed in each side chamber. Then, a stranger mouse (stranger 1, age-matched) was placed in the wired cup of any side chamber, and the other was left empty. The subject mouse was allowed to explore for 10 min. Time spent by the subject mouse in each chamber was video recorded, and its sociability was analyzed by a computer software. For social novelty preference test, another stranger (stranger 2, age-matched) was placed in the wired cup of the other side chamber. Time spent by the subject mouse for each stranger (strangers 1 and 2) was recorded for 10 min and then analyzed. The time spent with its head facing the cup from a distance of within 1 cm was regarded as time spent for this cup [8].

### Body weight, brain weight, and histological features of the prefrontal cortex

Both control and arsenite-F2 mice were weighed before the experiment. After completion of social behavior tests, the mice were sacrificed under deep pentobarbital anesthesia. The whole brain was collected and weighed by digital balance. At the age of 41 weeks, the prefrontal cortex from two mice each from the control group and four mice from the arsenite group was dissected, and tissues for histological study were fixed with neutral formalin 10%, embedded in paraffin, and then manually sectioned with a microtome to obtain 4–5μm-thick paraffin sections. Dewaxed sections are then stained with hematoxylin and eosin (H&E) for histological study under ordinary light microscope. Chromatolysis, gliosis, and necrosis were examined as common features of arsenic neurotoxicity [12].

### Quantification of social behavior-related gene expression

In the present study, the prefrontal cortex (*n* = 7 control mice and *n* = 8 arsenite mice from 41-week old and *n* = 7 control mice and *n* = 11 arsenite mice from 74-week old) collected for the gene expression assay was frozen in liquid nitrogen and then stored at – 80 °C. Total RNA extraction was performed by EZ-1 RNA tissue mini kits and BioRobot EZ-1 (Qiagen GmbH, Hilden, Germany) as described previously [13]. Purity and concentration of total RNA was examined by ND-1000 NanoDrop RNA Assay protocol (NanoDrop Technologies, Wilmington, DE, USA). Then, first strand cDNA synthesis from total RNA was done by SuperScript RNase H-Reverse Transcriptase II (Invitrogen, Carlsbad, CA, USA) and thermal cycler (Gene Atlas E, ASTEC, Fukuoka, Japan). The mRNA expression levels of 18S rRNA (internal control), 5-hydroxytryptamine (serotonin) receptor 5B (5-HT 5B), dopamine receptor D1a (Drd1a), and brain-derived neurotrophic factor (BDNF) were determined by using StepOne real-time PCR system (Applied Biosystems Inc., Foster City, CA, USA) in 41-week-old and 74-week-old groups of F2 male mice. The primers for 5-HT 5B (NM_024395), Drd1a (NM_010076), and BDNF (NM_012513) were purchased from Qiagen Sample & Assay Technologies. The primers for 18S rRNA (forward: 50-TACCACATCCAAAAGGCAG-30, reverse: 50-TGCCCTCCAATGGATCCTC-30) were purchased from Hokkaido System Science. Data were analyzed by using comparative threshold cycle method. The relative mRNA expression levels were expressed as mRNA signals per unit of 18S rRNA expression.

### Quantification of oxidative stress and inflammatory markers

The mRNA expression levels of heme oxygenase-1 (HO-1), interleukin (IL) 1β, and cyclooxygenase-2 (COX-2) were determined in both age groups of F2 male mice by using StepOne real-time PCR system (Applied Biosystems Inc., Foster City, CA, USA). The primers for HO-1 (NM_010442), IL-1β (NM_008361), and COX-2 (NM_011198) were purchased from Qiagen Sample and Assay Technologies. Data were analyzed as above.

### Measurement of plasma 8-hydroxy-2′-deoxyguanosine (8-OHdG)

The control and arsenic mice were sacrificed under pentobarbital anesthesia 24 h after completion of 3-chamber social behavioral test, and blood samples were collected (*n* = 6 for each group). Highly sensitive 8OHdG Check ELISA kit (Code #KOG-HS10E) was used to measure the plasma 8-OHdG levels according to the manufacturer’s (Nikken Seil Co., Ltd, Fukuroi, Shizuoka, Japan) instruction.

### Statistical analysis

All the data were presented as mean ± standard error (SE). Statistical Package for the Social Sciences (SPSS)-version 26 (IBM Corp., Armonk, NY) was used for statistical analysis. To detect the exploration time of the same subject in sociability and social novelty preference, paired *t* test was used between E and S1, and S1 and S2. To detect the messenger RNA levels, Student’s *t* test was used to analyze between the control and arsenite groups. Difference at *p* <0.05 was regarded as significant.

## Results

### Effect of maternal arsenic exposure on social behavior

**Fig. 2:**
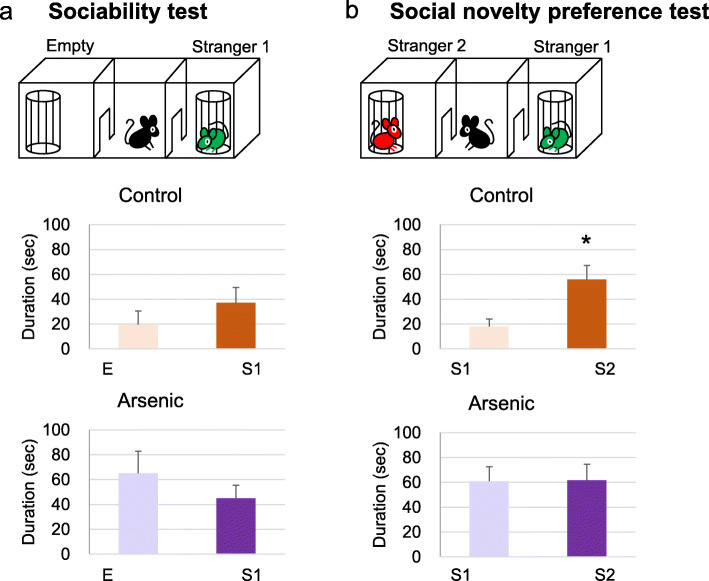
Social behavior test at 41-week-old. **a** Sociability test and **b** social novelty preference test in the control group and the arsenic group of 41-week-old F2 male mice. Each bar represents the mean ± SE (*n* = 9–12, *p < 0.05). E, empty; S1, stranger 1; S2, stranger 2

**Fig. 3:**
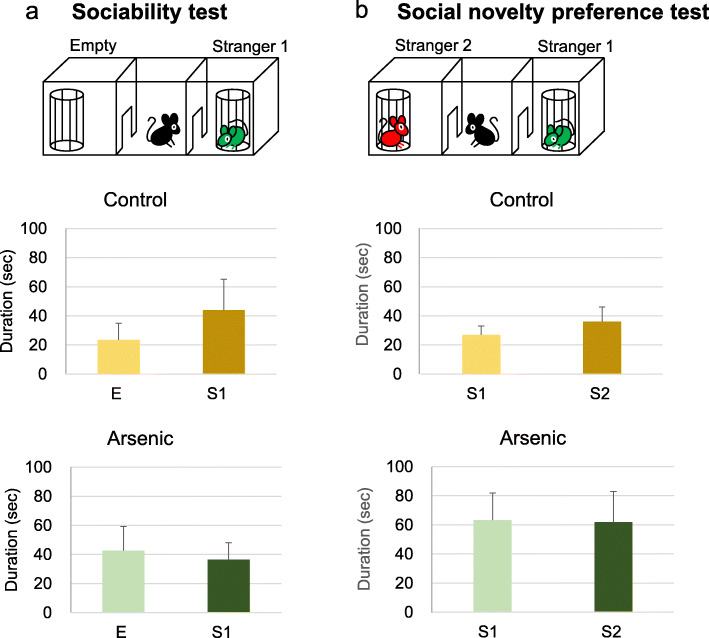
Social behavior test at 74-week-old. **a** Sociability test and **b** social novelty preference test in the control group and the arsenic group of 74-week-old F2 male mice. Each bar represents the mean ± SE (*n* = 9–15). E, empty; S1, stranger 1; S2, stranger 2

In control mice of the 41-week-old group, time spent for stranger 1 has a tendency to be greater than that for empty cup, but not statistically different. In the arsenite-F2 mice of the same group, time spent for stranger 1 was less, indicating poor sociability (Fig. 2). For social novelty preference test, the arsenite-F2 mice showed no difference between stranger 1 and stranger 2, while the control mice spent more time for stranger 2 significantly (*t* = 2.53, df = 19, *p* = 0.018) (Fig. 2). Similarly, in the 74-week-old group, the arsenite-F2 mice appeared with poor sociability and poor social novelty preference, but not statistically different (Fig. 3).

### Effect of maternal arsenic exposure on prefrontal cortex histological features

**Fig. 4:**
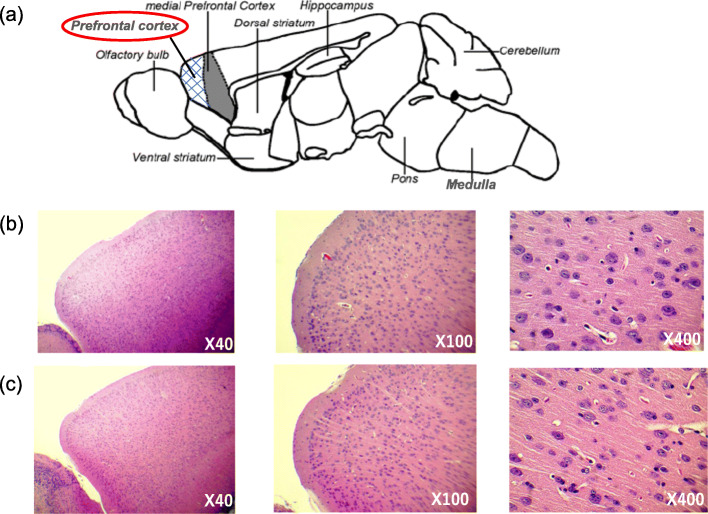
Mouse brain and representative histological photographs. **a** Mouse brain regions and prefrontal cortex of **b** tap water (control) and **c** tap water containing arsenic exposed 41-week-old F2 male mice. (H&E stain) (*n* = 2–4)

There was no difference between the control mice and the arsenite-F2 mice in comparing their body weight and brain weight. Histological features of the prefrontal cortex also showed no significant difference between the control mice of the 41-week old group and the arsenite-F2 mice of the 41-week-old group (Fig. 4). There were no features of chromatolysis, gliosis, and necrosis.

### Effect of maternal arsenic exposure on prefrontal cortex expression of social behavior-related genes

**Fig. 5:**
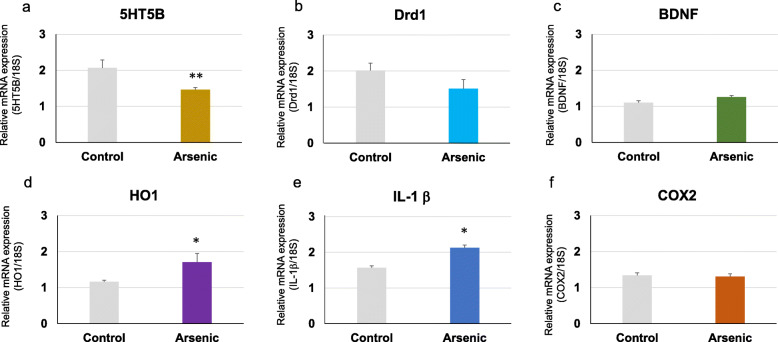
Messenger RNA expression level. **a** 5-HT 5B, **b** Drd1a, **c** BDNF, **d** HO-1, **e** IL-1β, and **f** COX-2 in the prefrontal cortex of the control group and the arsenic group of 41-week-old F2 male mice. Each bar represents the mean ± SE (*n* = 7–8, *p < 0.05, **p < 0.01)

**Fig. 6:**
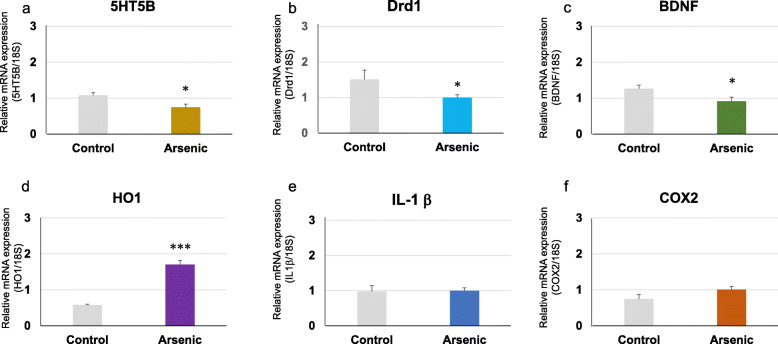
Messenger RNA expression level. **a** 5-HT 5B, **b** Drd1a, **c** BDNF, **d** HO-1, **e** IL-1β, and **f** COX-2 in the prefrontal cortex of the control group and the arsenic group of 74-week-old F2 male mice. Each bar represents the mean ± SE (*n* = 7–11, *p < 0.05, ***p < 0.001)

Regarding social behavior-related genes, we focussed to examine the serotonin receptor (5-HT 5B), dopamine receptor (Drd1a), and BDNF mRNAs in the prefrontal cortex. 5-HT 5B mRNA was significantly (*p* < 0.05) decreased in the arsenite-F2 mice compared to the control mice in both 41-week-old (*t* = 2.98, df = 19, *p* = 0.008) and 74-week-old groups (*t* = 2.74, df = 16, *p* = 0.014) groups (Figs. 5a and 6a). In addition, Drd1a mRNA tended to decrease in the arsenite-F2 mice in 41-week-old (*t* = 2.08, df = 15, *p* = 0.057) (Fig. 5b) and was significantly (*p* < 0.05) decreased in 74-week-old (*t* = 2.11, df = 16, *p* = 0.044) groups (Fig. 6b).

For BDNF gene expression, it was only significantly (*p* < 0.05) reduced in the arsenite-F2 mice of the 74-week-old group (*t* = 2.18, df = 16, *p* = 0.045) (Fig. 6c), but not in that of the 41-week-old group, compared to the respective control mice (Fig. 5c).

### Effect of maternal arsenic exposure on prefrontal cortex expression of oxidative stress and inflammatory marker genes

The oxidative stress marker HO-1 gene expression was significantly increased in the arsenite-F2 mice of both 41-week-old (*t* = −2.23, df = 11, *p* = 0.045) and 74-week-old (*t* = −7.89, df = 16, *p* = 0.0000007) groups compared to the control mice (Fig. 5d and 6d). Proinflammatory cytokine IL-1β was significantly (*p* < 0.05) increased in the arsenite-F2 mice in 41-week-old (*t* = 2.17, df = 16, *p* = 0.040) (Fig. 5e) and not significantly different in the arsenite-F2 mice in 74-week-old (*t* = 2.09, df = 15, *p* = 0.098) (Fig. 6e). The potent inflammatory marker COX-2 mRNA showed no significant difference between the arsenite-F2 and control mice in both groups (Fig. 5f and 6f).

### Effect of maternal arsenic exposure on plasma 8-OHdG levels

**Fig. 7:**
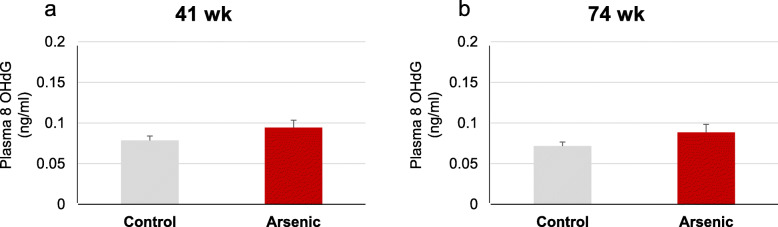
Plasma 8OHdG level in **a** 41-week-old and **b** 74-week-old F2 male mice. Each bar represents the mean ± SE (*n* = 6)

Plasma 8OHdG is a sensitive marker of oxidative DNA damage. We measured plasma 8OHdG levels, and tendency to increased plasma 8OHdG levels were observed in both 41- and 74-week-old arsenite F2 mice (Fig. 7 a and b)

## Discussion

The major findings of this study were impaired social behavior and decreased 5-HT 5B gene expression in the arsenite-F2 male mice. In our previous study of the F1 male mice, similar findings were observed, and we postulated that impaired social behavior might be due to deranged serotonergic system in the prefrontal cortex [8]. Prefrontal cortex is concerned with cognitive function and social behavior. Role of serotonergic system in the prefrontal cortex is neuromodulating effect, and it stabilizes the pyramidal cell excitation in the prefrontal cortex. It is important for proper social behavior and normal mood status. Neurotoxic effect of arsenic might involve the serotonergic system, resulting in poor sociability and social novelty preference.

In sociability test, the time spent for stranger 1 was not much different between the control and arsenite-F2 mice. The key difference was the time spent for empty cup; the arsenite-F2 mice tended to spend more time to empty cup. It could be interpreted as a kind of social isolation, and it was more distinct in the 41-week-old group. In social novelty preference test, the arsenite-F2 mice seemed to have no preference to either stranger 1 or stranger 2. These behavioral changes were clearly seen in both age groups. Recently, Valles et al. reported that altered motor activity and increased anxiety-like behaviors in zebrafish were transmitted to the F2 generation after ancestral exposure to arsenic in F0. They pointed out that arsenic was a neurotoxicant and potent epigenetic disruptor [14].

In the present study, the C3H male mice were used as a good model for social behavior assessment and neurochemical analysis [8, 15]. We studied the social behavior and the mRNA expressions related to behavior in two age groups of C3H mice; namely the 41-week-old group as a mature adult group and the 74-week-old group as an old adult group. Social behavior might be changed with age, and so we compared the social behavior between the two age groups. However, there was no significant difference between two age groups, regarding social behavior. Social behavior changes in arsenite-F2 mice were also similar to that of gestationally arsenite-exposed F1 mice in our previous study [8].

In the present study, sodium arsenite (NaAsO_2_, 85 ppm (85 mg/L) was given only to F0 pregnant mice from gestational days 8–18 (critical period of neurodevelopment). Since species differences were observed between human and mouse, a high dose is required for detection of neurotoxic effects in C3H mice. This is a standard dose to detect the effects of maternal exposure to arsenic on neurotoxicity, reproductive toxicity, and carcinogenicity in our research group [9, 10, 16]. No maternal toxicity and teratogenicity were observed at the dose of the present study. We only used the male pups because there is a sex difference in susceptibility to arsenic. Moreover, there is the oestrous cycle in female and possible hormonal effects on social behavior [17].

Neuronal morphology changes in the prelimbic cortex, a part of the prefrontal cortex, were studied by Aung et al. in 2016. They found that arsenic exposure was associated with a significant increase in the number of pyramidal neurons in layers V and VI of the prelimbic cortex. The prenatal arsenic exposure was associated with a significant decrease in neurite length but not dendrite spine density in all layers of the prelimbic cortex. In the present study, there were no significant histological changes, such as chromatolysis, gliosis, and necrosis, of the prefrontal cortex in the arsenite-F2 mice. It might be due to the absence of direct or developmental exposure to arsenic in F2 mice. Otherwise, immunohistochemical study would be helpful to detect more details than ordinary histological study using H&E stain.

Although there were no gross and histological changes in the prefrontal cortex, we found that the behavioral and neurochemical changes in the prefrontal cortex occurred in the arsenite-F2 mice. We studied the mRNA expressions of 5-HT 5B and BDNF in the prefrontal cortex, and these were found to be decreased in the arsenite-F2 mice compared to the control mice. These changes seemed to be inherited and might be the result of epigenetic disruption. In the present study, we could not prove that it was epigenetic transgenerational inheritance. However, these changes were found consistently in both gestationally arsenite-exposed F1 mice [6] and arsenite-F2 mice in the present study.

Since serotonin was known as a neuromodulator, we hypothesized that decreased serotonin receptors (5-HT 5B) would be responsible for glutamate-induced hyperexcitation of pyramidal cells and cell destruction in the prefrontal cortex. This led to impaired cognitive function, social memory, and finally social behavior. This effect was augmented by the decreased in BDNF which had a neuroprotective effect on many brain regions. Almeida and co-workers demonstrated the neuroprotective effect of BDNF against glutamate-induced apoptotic cell death in the hippocampus [18]. The decreased mRNA expression of BDNF in the prefrontal cortex was seen in the F1 of our previous study [6] and also in the old adult group of arsenite-F2 mice in the present study.

Arsenic-induced BDNF decrease was convinced by both animal and human studies. Valles et al. found that a reduction in BDNF gene expression in the F0 and F2 generation of zebrafish was induced by inorganic arsenic exposure [14]. In a human study, Karim and co-workers described that serum BDNF level was significantly (*p*<0.001) decreased in people living in arsenic-endemic area, and there was a dose-response relationship between arsenic exposure and serum BDNF levels [19]. Role of BDNF was not only as a neuroprotective agent, but also as a synaptic regulator in the brain. Its synaptogenic and synaptoplastic effects in the prefrontal cortex were also important for cognitive function, and there was an interaction between BDNF and serotonin regarding social behavior.

In addition to 5-HT 5B and BDNF, we also studied the mRNA expression of dopamine receptor in the prefrontal cortex. Dopamine is one of the major markers in social brain, and among the dopamine receptors, dopamine D1 receptor (Drd1) plays a major role in social cognition [20]. In the present study, decreased Drd1a gene expression was observed in both age groups of arenite-F2 mice, significantly in the old adult group. This finding was also supportive to the occurrence of impaired social behavior in the arsenic group. It was consistent with the findings of Homberg and colleagues. In their study, they stated that sociability and social novelty preference were significantly reduced in the Drd1^I116S^ mutant rats compared to wild-type rats [20].

We also investigated the oxidative stress and inflammatory markers, HO-1 and COX-2, because almost all arsenic effects were based on oxidative stress and tissue inflammation [2]. In both mature adult group and old adult group of arsenite-F2 mice, mRNA expression of HO-1 was significantly increased. Increased HO-1 expression was associated with a variety of conditions, including ultraviolet irradiation, hyperthermia, inflammatory cytokines, heavy metals, apoptosis, and cancers [21]. In the present study, the oxidative stress indicated by HO-1 expression might not be due to direct effect of sodium arsenite because F2 mice were never exposed to arsenic directly. It might be probably due to glutamate-induced apoptotic cell death in the prefrontal cortex as discussed above.

Otherwise, we could not exclude the other possible conditions of upregulation of HO-1 in this study because HO-1 is a non-specific oxidative stress marker. To explore more information on oxidative stress, we studied plasma 8OHdG which is a sensitive marker of oxidative DNA damage [22]. In the present study, there was no significant increase in plasma 8OHdG level in arsenite-F2 mice. These findings suggested that increased HO-1 was not likely due to oxidative DNA damage and apoptotic cell death. HO-1 response might be due to other non-specific inflammatory process.

The IL-1β is a potent inflammatory cytokine which is important for regulation of other inflammatory cytokines such as IL-6 and IL-8 [23]. In the present study, IL-1β mRNA expression was only increased in 41-week-old arsenite-F2 mice. Regarding COX-2, it was a potent inflammatory marker, and we did not detect its upregulation as HO-1 in this study. Similar to HO-1, COX-2 was also associated with inflammation, apoptosis, and cancers [24]. In contrast, COX-2 mediates inflammation while HO-1 modulates it. The HO-1 has antioxidant, anti-inflammatory, antiapoptotic, and antiproliferative effects [25]. The overexpression of COX-2 induces HO-1 expression which in turn inhibits COX-2 expression [26]. Therefore, in the present study, there was an increase in HO-1 expression, but not in COX-2 expression.

## Conclusions

Exposure to sodium arsenite (85 ppm in drinking water) during GD 8-18 in C3H pregnant mice (F0) was responsible for social behavioral changes in both 41-week-old and 74-week-old F2 male mice. Social behavior is mainly concerned with the prefrontal cortex and so we studied the histological features and social behavioral-related gene expression in the prefrontal cortex. We hypothesized that decreased serotonin receptors (5-HT 5B) impaired the neuromodulating effect of serotonergic system on pyramidal cells, resulting in glutamate-induced hyperexcitation and apoptotic cell death in the prefrontal cortex. In addition, neuroprotective effect of BDNF was decreased, and cognitive function, social memory, and social behavior were impaired. Reduction in dopamine receptor (Drd1a) gene expression was also a relevant finding concerned with impaired social cognition. Although there were indicative findings of oxidative stress (HO-1) and inflammatory reactions (IL-1β), we failed to provide the evidence of apoptotic cell death in histological study. There was also no significant increase in plasma level of oxidative DNA damage marker (8-OHdG). However, we could demonstrate that decreased expressions of 5-HT 5B and BDNF were neurotoxic effect of arsenic which passed to F2 male mice born to gestationally arsenic-exposed F1 mice. Finally, it could be concluded that the social behavior changes due to neurotoxic effect of arsenic could be observed in F2 male mice without direct exposure to sodium arsenite.

## Data Availability

The data of current study are available from the corresponding author on reasonable request.

## Abbreviations

F1: First generation
F2: Second generation
5-HT 5B: 5-hydroxytryptamine (serotonin) receptor 5B
Drd1a: Dopamine receptor D1a
BDNF: Brain-derived neurotrophic factor
COX-2: Cyclooxygenase 2
HO1: Heme oxygenase 1
IL-1β: Interleukin-1β
8OHdG: 8-hydroxy-2′-deoxyguanosine
NaAsO_2_: Sodium arsenite
